# An Integrated Fluid-Chemical Model Toward Modeling the Formation of Intra-Luminal Thrombus in Abdominal Aortic Aneurysms

**DOI:** 10.3389/fphys.2012.00266

**Published:** 2012-07-20

**Authors:** Jacopo Biasetti, Pier Giorgio Spazzini, Jesper Swedenborg, T. Christian Gasser

**Affiliations:** ^1^Department of Solid Mechanics, School of Engineering Sciences, KTH Royal Institute of TechnologyStockholm, Sweden; ^2^Mechanics Division, National Institute of Metrological ResearchTurin, Italy; ^3^Department of Molecular Medicine and Surgery, Center for Molecular Medicine, Karolinska InstitutetStockholm, Sweden

**Keywords:** abdominal aortic aneurysm, coagulation cascade, computational fluid dynamics, convection-diffusion-reaction equations, intra-luminal thrombus, platelets, thrombin, vortical structures

## Abstract

Abdominal Aortic Aneurysms (AAAs) are frequently characterized by the presence of an Intra-Luminal Thrombus (ILT) known to influence their evolution biochemically and biomechanically. The ILT progression mechanism is still unclear and little is known regarding the impact of the chemical species transported by blood flow on this mechanism. Chemical agonists and antagonists of platelets activation, aggregation, and adhesion and the proteins involved in the coagulation cascade (CC) may play an important role in ILT development. Starting from this assumption, the evolution of chemical species involved in the CC, their relation to coherent vortical structures (VSs) and their possible effect on ILT evolution have been studied. To this end a fluid-chemical model that simulates the CC through a series of convection-diffusion-reaction (CDR) equations has been developed. The model involves plasma-phase and surface-bound enzymes and zymogens, and includes both plasma-phase and membrane-phase reactions. Blood is modeled as a non-Newtonian incompressible fluid. VSs convect thrombin in the domain and lead to the high concentration observed in the distal portion of the AAA. This finding is in line with the clinical observations showing that the thickest ILT is usually seen in the distal AAA region. The proposed model, due to its ability to couple the fluid and chemical domains, provides an integrated mechanochemical picture that potentially could help unveil mechanisms of ILT formation and development.

## 1 Introduction

Focal dilatations of the abdominal aorta, known as Abdominal Aortic Aneurysms (AAAs), are frequently observed in the aging male population (Fleming et al., 2005) and their rupture is fatal in up to 90% of cases (Upchurch and Schaub, 2006). Rupture is prevented through elective surgical intervention. Clearly, the ability to predict lesions that are at risk is required to optimize medical and economic outcomes.

AAAs are thought to be the end results of an irreversible pathological remodeling of the extracellular matrix due to an excessive proteolytic activity (Choke et al., 2005; Michel et al., 2011); in particular, loss of structural integrity of the major ground substances elastin and collagen. A thin or thick Intra-Luminal Thrombus (ILT) is a tissue found in nearly all AAAs large enough to indicate risk of rupture (Hans et al., 2005). While the thin ILT is not explored very well, the thick ILT (Folkesson et al., 2011) has solid-like properties (Gasser et al., 2008) and is composed of a fibrin mesh, traversed by a continuous network of interconnected canaliculi (Swedenborg and Eriksson, 2006), incorporated with blood cells, e.g., erythrocytes and neutrophils, aggregated platelets, blood proteins, and cellular debris. The ILT is thought to play an important role in the pathology and natural history of AAAs with a series of effects on the underlying aortic wall. Specifically it causes localized hypoxia, possibly leading to increased neovascularization, inflammation, and local wall weakening (Vorp et al., 2001). In addition, the changes to matrix-degrading protease expression (Kazi et al., 2005) and structural and cellular composition (Kazi et al., 2003) lead to a thinner wall compared to the aneurysm wall exposed to flowing blood (Swedenborg and Eriksson, 2006). The ILT has a significant structural impact on the biomechanics of AAAs and influences both the magnitude and the distribution of wall stress (Inzoli et al., 1993; di Martino et al., 1998; Wang et al., 2002; Polzer et al., 2011) and needs to be considered through a biomechanical rupture risk assessment (Gasser et al., 2010).

The hemostatic system maintains the integrity of the circulatory system in case of vascular damage. It maintains blood in a fluid state and responds to vessel injury by the rapid formation of a clot. The clot formation is the end result of a process initiated by the injury of a vessel wall and subsequent exposure of the subendothelium to blood flow. This triggers two interconnected processes: platelets (PLTs) aggregation and the CC. The first process involves platelets, anucleated cells that originate from bone marrow and circulate in blood as sentinels of vascular integrity. Platelets can be activated and adhere to the sites of exposed subendothelium. During this process the shape of the platelets modifies and chemicals are released in the blood stream so that new platelets are activated leading to aggregation with free or wall-bound ones.

The CC consists of a series of enzymatic reactions, in which a series of proenzymes (zymogens) is turned into their active enzyme form. The CC is triggered as soon as tissue factor (TF) binds to blood-borne factor VIIa. The series of activated reactions lead to the formation of thrombin, which in turn converts fibrinogen into fibrin (Gailani and Renné, 2007; Mackman et al., 2007). This process can lead to three outcomes: the impairment of clotting leading to bleeding disorders (like hemophilia), hemostasis, or hypercoagulability that in turn lead to thrombotic events such as the formation of an ILT. Platelet activation, aggregation, and adhesion can be regarded as the initiating response of thrombus formation that arrests hemorrhage in response to vascular injury and permits wound healing (Ruggeri and Mendolicchio, 2007), whereas actual hemostasis requires both platelets and the CC to occur.

It has long been known (Bluestein et al., 1996) that blood dynamics has a relevant influence on the clotting processes, consequently a meaningful ILT formation model should account for the biochemical and biomechanical interactions as it has been proposed by different studies in the literature (Hubbell and McIntire, 1986; Folie and McIntire, 1989; Kuharsky and Fogelson, 2001; Fogelson and Tania, 2005; Xu et al., 2008). However, simplistic geometrical, hemodynamical, and sometimes even biochemical, assumptions were applied. On the other hand, works such as Xu et al. (2008), despite the aforementioned limitations, present a multiscale approach able to integrate also the discrete blood elements and, therefore, are fundamental for a microscopic description of the coagulation process. The present work considers biochemical and biomechanical factors in the abdominal aorta through a macroscopic model of the CC and the Navier–Stokes equations, respectively. Specifically, the coupling is obtained by a set of convection-diffusion-reaction (CDR) equations added on top of the computed blood flow field. The proposed model predicts the distribution of chemicals in complex AAA blood flows and hence has the potential to improve our current understanding of ILT pathophysiology.

## 2 Materials and Methods

### 2.1 Geometry representation and discretization

**Figure 1:**
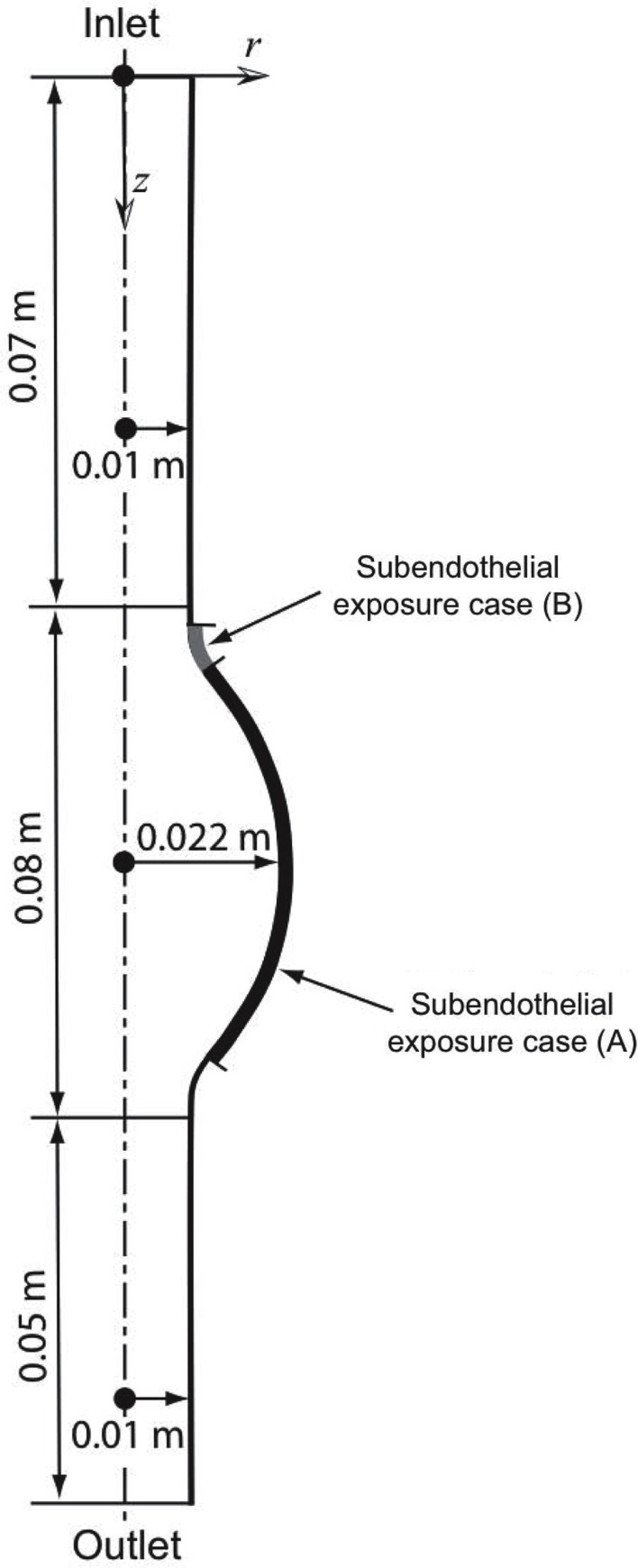
**Axisymmetric fusiform abdominal aortic aneurysm (AAA) of 4.4 cm in (luminal) diameter with the dotted line denoting the axis of symmetry**. Two cases of exposed subendothelium were introduced: Case (A) considers a large exposure and Case (B) a focal exposure, see also Section 2.2.3.

An idealized 2D-axisymmetric AAA has been modeled to simulate a small fusiform aneurysm of 4.4 cm in (luminal) diameter, a size of clinical relevance sufficiently large to alter blood flow (Figure 1). We consider a thin-ILT-covered wall (Figure 3), a typical feature of AAA progression (Swedenborg, J. Personal Communication), with two regions of exposed subendothelium: Case (A) considers a large exposed area and Case (B) a focal one (Figure 1). The two cases aim at investigating the effect of varying size and position of the exposed subendothelium.

**Figure 2:**
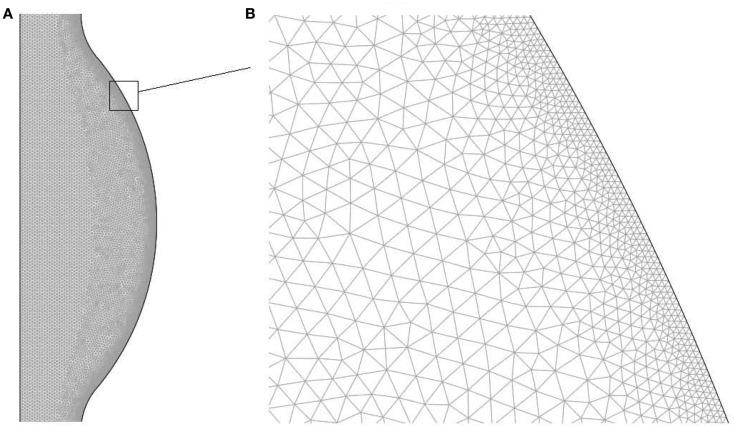
**Finite element mesh used to solve the fluid dynamical and chemical set of equations: (A) bulge region, (B) zoom of the near wall region**.

The domain has been discretized with an unstructured triangular mesh with higher density within concentration and velocity boundary layers. Details regarding the mesh are presented in Table 1 and in Figure 2 a region of the mesh is shown. The mesh was generated using the Advancing Front Method (Thompson et al., 1998) provided by COMSOL v3.5a (COMSOL AB).

**Table 1:** **FE mesh data**.

No. of elements	DoF fluid	DoF CDR	Element quality *q*
58,194	268,401	547,254	>0.81

*Degrees of freedom is denoted by DoF, and the quality of the triangular elements is given as $q=\frac{4\sqrt{3}A}{h_{1}^{2}+h_{2}^{2}+h_{3}^{2}}$ (COMSOL, 2008), where *A* and *h_i_*, *i *= 1, 2, 3 denote element area and edge lengths, respectively*.

A mesh convergence study verified that the computed results were mesh independent. For the fluid dynamical problem the velocity, pressure, and wall shear stress fields were sampled at different locations and times during the cardiac cycle (see Prakash and Ethier, 2001). For the CDR problem the concentration of thrombin, which is the final product of the modeled chemical reactions, was sampled.

### 2.2 Mathematical models and solution procedure

#### 2.2.1 Fluid dynamical model

The fluid dynamical field was rendered by the continuity and Navier–Stokes equations,

$$\begin{array}{llll} & \nabla \cdot \text{u}=0, & & \text{(1)}\\ & \rho \left(\frac{\partial \text{u}}{\partial t}+\text{u}\cdot \nabla \text{u}\right)=-\nabla p+\nabla \cdot \tau , & & \text{(2)} \end{array}$$

where **u** is the velocity vector, ρ the density, *p* the pressure, and **τ** = 2μ**D** the deviatoric viscous stress tensor (Acheson, 2003). Boundary conditions, as previously used (Biasetti et al., 2010, 2011), were applied. At time *t* the inlet mass flow rate was prescribed and the inlet velocity profile **u** was calculated according to the no-slip boundary condition and the Navier–Stokes equations. At the outlet a prescribed pressure waveform with no-viscous stress boundary condition was applied. The prescribed waveform takes the form of *p *= *p_t_*(*t*), where *p_t_*(*t*) is a tabulated set of pressures in time, whilst the no-viscous stress condition prescribes that **τn **= **0**. In the case of rigid wall and incompressible fluid the imposition of a pressure wave at the outlet is not strictly necessary since given the mass flow rate, the pressure differential (Δ*p*) is calculated. This means that a constant pressure may also be applied. However, in order to get a meaningful pressure distribution (amplitude and phase) a physiological pressure wave need to be used. For a detailed explanation of different boundary conditions the reader is referred to Vignon-Clementel et al. (2006). The no-slip boundary condition was applied at the walls and at the symmetry axis an axial symmetry condition was set.

#### 2.2.2 Constitutive modeling of blood

Blood has complex rheological properties involving shear-thinning, thixotropy, and viscoelasticity (Oka, 1981; McDonald, 2004). Its non-Newtonian behavior has recently been shown (Biasetti et al., 2011) to produce substantial differences in the local flow pattern compared to a Newtonian viscosity model; as such differences are mostly confined on a local level, a model dealing with local phenomena like platelets’ dynamics and chemicals’ concentrations should take the non-Newtonian behavior into proper consideration. We adopted the Carreau-Yasuda (CY) model for shear-thinning (Bird et al., 1987)

(3) $$\frac{\mu -\mu_{\infty }}{\mu_{0}-\mu_{\infty }}={\left[1+{\left(\lambda \overset{{}^{\circ}}{\gamma }\right)}^{a}\right]}^{\frac{n-1}{a}},$$

where $\overset{{}^{\circ}}{\gamma }=\sqrt{2\text{D}:\text{D}}$ denotes the scalar shear rate with **D **= (***l* **+ ***l***^T^)/2 being the symmetric part of the velocity gradient tensor ***l*** (Malvern, 1969). The antisymmetric part **Ω** = (***l* **− ***l***^T^)/2 of ***l***, as it is used in Section 3, was also introduced. Note that at the lower and upper ends of the shear rate spectrum equation (3) describes blood as a Newtonian fluid of viscosities μ_0_ and μ_∞_, whereas the parameters λ, *n*, and *a* define the transition between these asymptotic conditions. Table 2 reports the Carreau-Yasuda model parameters applied in the present work, which were selected to fit experimental data of blood at 37.0°C (Leuprecht and Perktold, 2001; Abraham et al., 2005).

**Table 2:** **Carreau-Yasuda constitutive parameters to model blood at 37.0°C**.

μ_0_	μ_∞_	λ	*n*	*a*	ρ
0.16 Pa s	0.0035 Pa s	8.2 s	0.2128	0.64	1050 kg/m^3^

#### 2.2.3 The coagulation cascade to thrombin formation

**Figure 3:**
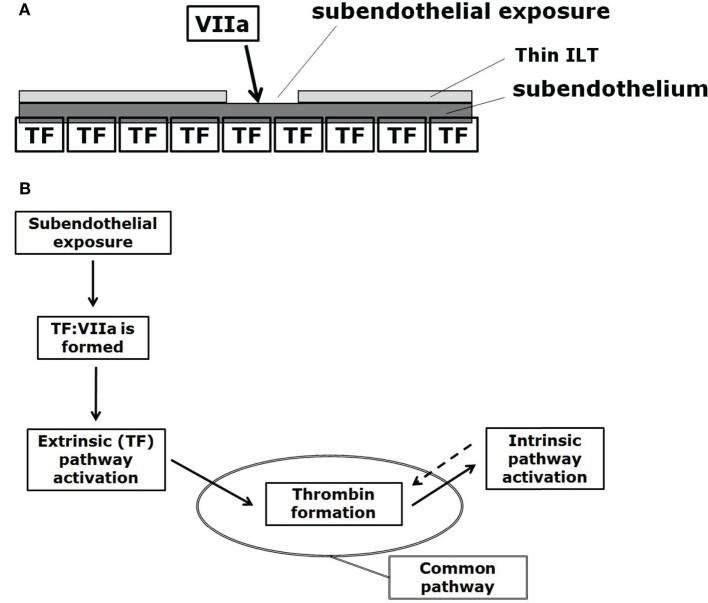
**(A)** Initiation of the CC at sites of subendothelial exposure, i.e., when extravascular tissue factor (TF) binds with blood-borne factor VIIa. **(B)** CC: subendothelial exposure triggers thrombin formation via the extrinsic (Tissue Factor, TF) pathway. Positive feedback (denoted by the dotted arrow) of the intrinsic pathway increases thrombin formation. The reaction from prothrombin to thrombin formally belongs to the common pathway, here indicated by the oval.

The CC is a series of subsequent reactions, where the activation of one clotting factor activates the ensuing one, finally leading to thrombin generation. The CC is triggered at sites of endothelial damage, i.e., when extravascular tissue factor (TF), which is present under the endothelial layer, binds with blood-borne factor VIIa (Figure 3A).

The CC can be divided into the *extrinsic*, the *intrinsic* (or *contact*), and the *common* pathways (Figure 3B). The extrinsic pathway is also referred as the *tissue factor* pathway and it is thought to be the primary activator of the CC (at least for cases where a foreign surface is not involved for which the intrinsic pathway is thought to start the CC; Basmadjian et al., 1997) while being also the essential one, meaning that without its reactions the CC cannot start (Hoffman and Monroe, 2007; Mackman et al., 2007).

We followed the CC model proposed in Jones and Mann (1994) and empirically supported by a companion work (Stram et al., 1994). It consists of 18 species and involves plasma-phase and surface-bound enzymes and zymogens (see Table 3) where the subscript “a” denotes the active complex. The model includes both plasma-phase and membrane-phase reactions. Note that for the present CC model, tissue factor (TF), and factor VIIa are not considered separately but they are already joined to form TF:VIIa.

**Table 3:** **Proenzymes and enzymes involved in the CC model**.

Inactive	Active
IX (Antihemophilic factor B or Christmas factor)	IXa
X (Stuart-Prower factor)	Xa
V (Proaccelerin)	Va
VIII (Antihemophilic factor)	VIIIa
II (Prothrombin)	IIa (Thrombin)

A time-dependent version of the CC model has been implemented, where the 16 chemical reactions read:

(4) $$\text{IX}+\text{TF}:\text{VIIa}\overset{k_{6}}{\underset{k_{16}}{⇌}}\text{IX}:\text{TF}:\text{VIIa}\overset{k_{11}}{\to }\text{TF}:\text{VIIa}+\text{IXa},$$

(5) $$\text{X}+\text{TF}:\text{VIIa}\overset{k_{6}}{\underset{k_{17}}{⇌}}\text{X}:\text{TF}:\text{VIIa}\overset{k_{12}}{\to }\text{TF}:\text{VIIa}+\text{Xa},$$

(6) $$\text{X}+\text{VIIIa}:\text{IXa}\overset{k_{6}}{\underset{k_{18}}{⇌}}\text{X}:\text{VIIIa}:\text{IXa}\overset{k_{13}}{\to }\text{VIIIa}:\text{IXa}+\text{Xa},$$

$$\begin{array}{llll} & \text{IX}+\text{Xa}\overset{k_{15}}{\underset{}{\to }}\text{Xa}+\text{IXa}, & & \text{(7)}\\ & \text{V}+\text{Xa}\overset{k_{1}}{\underset{}{\to }}\text{Xa}+\text{Va}, & & \text{(8)}\\ & \text{VIII}+\text{Xa}\overset{k_{3}}{\underset{}{\to }}\text{Xa}+\text{VIIIa}, & & \text{(9)}\\ & \text{V}+\text{IIa}\overset{k_{2}}{\underset{}{\to }}\text{IIa}+\text{Va}, & & \text{(10)}\\ & \text{VIII}+\text{IIa}\overset{k_{4}}{\underset{}{\to }}\text{IIa}+\text{VIIIa}, & & \text{(11)} \end{array}$$

(12) $$\text{II}+\text{Va}:\text{Xa}\overset{k_{6}}{\underset{k_{19}}{⇌}}\text{II}:\text{Va}:\text{Xa}\overset{k_{14}}{\to }\text{Va}:\text{Xa}+\text{mIIa},$$

$$\begin{array}{llll} & \text{mIIa}+\text{Va}:\text{Xa}\overset{k_{5}}{\underset{}{\to }}\text{Va}:\text{Xa}+\text{IIa}, & & \text{(13)} \end{array}$$

(14) $$\text{VIIIa}+\text{IXa}\overset{k_{7}}{\underset{k_{9}}{⇌}}\text{VIIIa}:\text{IXa},$$

(15) $$\text{Va}+\text{Xa}\overset{k_{8}}{\underset{k_{10}}{⇌}}\text{Va}:\text{Xa}.$$

Despite more recent models include also the stoichiometric (tissue factor pathway inhibitor (TFPI) and antithrombin) and dynamic (dynamic protein C (PC) system) inhibitory processes (see for example Hockin et al., 2002), AAA pathology motivates the choice of the CC model (4)–(15). In the vasculature TFPI is mainly bound to the endothelium (Österund, 2012), which is to a large extent not present in AAAs. However, TFPI is also present in blood at a very low concentration and may still play a role in inhibiting the clotting process. For the same reason, i.e., lack of endothelium, the dynamic protein C (PC) system has not been considered in the present model since it needs, in order to be activated, the integral membrane protein thrombomodulin which is expressed on the surface of endothelial cells. Antithrombin is a relatively inefficient inhibitor on its own and needs to bind to heparan sulfate proteoglycans expressed on the vascular endothelium or heparin in order to accelerate its function (Rosenberg and Bauer, 1994). Heparan sulfate proteoglycans are not available due to the lack of endothelium and the effect of heparin is debated (Marcum et al., 1986). These reasons led to the exclusion of this contribution.

To show the reaction kinetics behavior in a simple case, the chemical reactions in equations (4)–(15) were first computed for the case of a perfectly mixed physical environment with constant volume, i.e., in a *batch* reactor, and at a constant temperature of 37°C. Initially all chemical species are at physiological concentration, see Table 4.

**Table 4:** **Initial concentration of the species involved in the CC model to thrombin formation**.

Species	Concentration (mol/m^3^)
IX	90E-6
TF:VIIa	1E-6
X	170E-6
V	20E-6
VIII	0.7E-6
VIIIa	100E-9
II	1.4E-3
IX:TF:VIIa	0
IXa	0
X:TF:VIIa	0
Xa	0
VIIIa:IXa	0
X:VIIIa:IXa	0
Va	0
IIa	0
Va:Xa	0
II:Va:Xa	0
mIIa	0

*All species are at physiological concentration (see Jones and Mann, 1994; Stram et al., 1994)*.

Introducing a concentration vector **c** ([mol/m^3^])^1^, the governing equation of the CC model reads

(16) $$\frac{\text{d}c}{\text{d}t}=\text{Sr},$$

where **S** denotes the stoichiometric matrix and **r** is the reaction rate vector ([mol/(m^3^ s)])^2^. Specifically, **S** is a 18 × 16 matrix that reads

$$\left[\;\begin{array}{cccccccccccccccc} -1 & 0 & 0 & 0 & 0 & 0 & -1 & 0 & 0 & 0 & 0 & 0 & 0 & 0 & 0 & 0\\ -1 & 1 & -1 & 1 & 0 & 0 & 0 & 0 & 0 & 0 & 0 & 0 & 0 & 0 & 0 & 0\\ 1 & -1 & 0 & 0 & 0 & 0 & 0 & 0 & 0 & 0 & 0 & 0 & 0 & 0 & 0 & 0\\ 0 & 1 & 0 & 0 & 0 & 0 & 1 & 0 & 0 & 0 & 0 & 0 & 0 & 0 & -1 & 0\\ 0 & 0 & -1 & 0 & -1 & 0 & 0 & 0 & 0 & 0 & 0 & 0 & 0 & 0 & 0 & 0\\ 0 & 0 & 1 & -1 & 0 & 0 & 0 & 0 & 0 & 0 & 0 & 0 & 0 & 0 & 0 & 0\\ 0 & 0 & 0 & 1 & 0 & 1 & 0 & 0 & 0 & 0 & 0 & 0 & 0 & 0 & 0 & -1\\ 0 & 0 & 0 & 0 & -1 & 1 & 0 & 0 & 0 & 0 & 0 & 0 & 0 & 0 & 1 & 0\\ 0 & 0 & 0 & 0 & 1 & -1 & 0 & 0 & 0 & 0 & 0 & 0 & 0 & 0 & 0 & 0\\ 0 & 0 & 0 & 0 & 0 & 0 & 0 & -1 & 0 & -1 & 0 & 0 & 0 & 0 & 0 & 0\\ 0 & 0 & 0 & 0 & 0 & 0 & 0 & 1 & 0 & 1 & 0 & 0 & 0 & 0 & 0 & -1\\ 0 & 0 & 0 & 0 & 0 & 0 & 0 & 0 & -1 & 0 & -1 & 0 & 0 & 0 & 0 & 0\\ 0 & 0 & 0 & 0 & 0 & 0 & 0 & 0 & 1 & 0 & 1 & 0 & 0 & 0 & -1 & 0\\ 0 & 0 & 0 & 0 & 0 & 0 & 0 & 0 & 0 & 0 & 0 & 0 & 0 & 1 & 0 & 0\\ 0 & 0 & 0 & 0 & 0 & 0 & 0 & 0 & 0 & 0 & 0 & -1 & 0 & 0 & 0 & 0\\ 0 & 0 & 0 & 0 & 0 & 0 & 0 & 0 & 0 & 0 & 0 & -1 & 1 & 0 & 0 & 1\\ 0 & 0 & 0 & 0 & 0 & 0 & 0 & 0 & 0 & 0 & 0 & 1 & -1 & 0 & 0 & 0\\ 0 & 0 & 0 & 0 & 0 & 0 & 0 & 0 & 0 & 0 & 0 & 0 & 1 & -1 & 0 & 0 \end{array}\;\right]\;,$$

where reversible reactions are considered through single reactions. The 16 columns in **S** represent the 16 chemical reactions in equations (4)–(15) that build up the model and **c** stores the concentration of the species in the following order: IX, TF:VIIa, IX:TF:VIIa, IXa, X, X:TF:VIIa, Xa, VIIIa:IXa, X:VIIIa:IXa, V, Va, VIII, VIIIa, IIa, II, Va:Xa, II:Va:Xa, mIIa. The rate constants, used in the CC model, are given in Table 5.

**Table 5:** **Rate constants used in the CC model (see Jones and Mann, 1994)**.

	Value	Units	Description
*k*_1_	2e4	mol^−1^ m^3^ s^−1^	Activation of V by Xa (2nd order)
*k*_2_	2e4	mol^−1^ m^3^ s^−1^	Activation of V by IIa (2nd order)
*k*_3_	1e4	mol^−1^ m^3^ s^−1^	Activation of VIII by Xa (2nd order)
*k*_4_	2e4	mol^−1^ m^3^ s^−1^	Activation of VIII by IIa (2nd order)
*k*_5_	1e4	mol^−1^ m^3^ s^−1^	Conversion of mIIa to IIa by Va:Xa (2nd order)
*k*_6_	1e5	mol^−1^ m^3^ s^−1^	On-rate for rapidly formed complexes (2nd order)
*k*_7_	1e4	mol^−1^ m^3^ s^−1^	On-rate for the VIIIa:IXa complex (2nd order)
*k*_8_	4e5	mol^−1^ m^3^ s^−1^	On-rate for the Va:Xa complex (2nd order)
*k*_9_	0.005	s^−1^	Off-rate for VIIIa:IXa complex
*k*_10_	0.4	s^−1^	Off-rate for Va:Xa complex
*k*_11_	0.3	s^−1^	Vmax for activation of IX by TF:VIIa (Michaelis–Menten kinetics)
*k*_12_	1.15	s^−1^	Vmax for activation of X by TF:VIIa (Michaelis–Menten kinetics)
*k*_13_	8.2	s^−1^	Vmax for activation of IX by VIIIa:IXa (Michaelis–Menten kinetics)
*k*_14_	32	s^−1^	Vmax for mIIa formation by Va:Xa (Michaelis–Menten kinetics)
*k*_15_	1e2	mol^−1^ m^3^ s^−1^	Activation of IX by Xa (2nd order)
*k*_16_	24	s^−1^	Off-rate for IX on TF:VIIa complex
*k*_17_	44	s^−1^	Off-rate for X on TF:VIIa complex
*k*_18_	0.001	s^−1^	Off-rate for X on VIIIa:IXa complex
*k*_19_	70	s^−1^	Off-rate for II on Va:Xa complex

*Michaelis–Menten kinetics (Metiu, 2006) models enzyme kinetics*.

#### 2.2.4 Convection-diffusion-reaction model

In the case of the *batch* reactor, chemical concentrations can be considered as a function of time only, because of the so called well-mixed assumption. On the other hand, in blood flows, including flows in AAAs, the concentrations vary in space and time, leading therefore to a 3D time-dependent problem. This can be solved by coupling the fluid dynamical model (see Section 2.2.1) to the time-dependent CC model (see Section 2.2.3). Specifically, the coupled model defines a set of evolution equations for species’ concentrations *c_i_*, *i* = 1, …, 18 that takes the form of a CDR problem and reads

$$\begin{array}{llll} & \underset{time\;\;dependence\;\;term}{\underset{︸}{\frac{\partial c_{i}}{\partial t}}}+\overset{diffusion\;\;term}{\overset{︷}{\nabla \cdot \left(-{\text{D}}_{i}\nabla c_{i}\right)}}=\underset{reaction\;\;term}{\underset{︸}{R_{i}}}-\overset{convective\;\;term}{\overset{︷}{u\cdot \nabla c_{i}}}, & & \\ & \;\;i=1,\ldots ,18, & & \text{(17)} \end{array}$$

where *R_i_* are the reaction terms (defined by the CC model, see Section 2.2.3), **u** the blood velocity vector (defined by the Navier–Stokes equations, see Section 2.2.1) and D*_i_* the isotropic diffusion coefficients. Values for D*_i_* were taken from Folie and McIntire (1989), Xu et al. (2008) and set equal, slightly modified (see Section 4), to 10^−8^ m^2^/s. Chemical species’ concentrations at the inlet are set equal to the values in Table 4 except for TF:VIIa which is set equal to zero. At sites of subendothelial exposure TF:VIIa is set equal to 1 × 10^−6^ mol/m^3^. The domain is initialized with the inlet values. The final CDR problem is clearly convection-dominated and requires numerical regularization as detailed in Section 2.2.5. Despite TF:VIIa, IX:TF:VIIa, and X:TF:VIIa are bound to the subendothelium, we model them also in the fluid domain by adding CDR equations for these three compounds. This choice is motivated by the fact that blood contains microparticles (MPs) that can express TF, allowing the formation of the TF:VIIa, IX:TF:VIIa, and X:TF:VIIa compounds (Owens and Mackman, 2011). These three compounds are not present in blood at the beginning of the simulation (see Section 3.2) but progressively appear from the site of exposed subendothelium, which is thought to model the gradual appearance of TF-bearing MPs from activated blood elements. Moreover, considering an intravascular source of TF is crucial in thrombosis, and in particular under conditions of low flow and impaired endothelium (Del Conde et al., 2005; Langer et al., 2008; Antoniak et al., 2009; Morel et al., 2009; Mann, 2011). Finally, it is noted that the present CC model is based on the assumption of an infinite supply of platelets, which in turn leads to an infinite binding site density. This assumption is satisfied in our computational model due to the continuous supply of fresh blood from upstream. For a discussion on the implications of a finite density of binding sites the reader is referred, for example, to Kuharsky and Fogelson (2001).

#### 2.2.5 Finite element discretization and numerical algorithms

##### 2.2.5.1 Time-dependent solution of the CC model

To solve the time-dependent CC model (see Section 2.2.3), the IDA solver for Differential-Algebraic Equation (DAE) systems (Hindmarsh et al., 2005), as implemented in COMSOL Reaction Engineering Lab v3.5a (COMSOL AB), was used.

##### 2.2.5.2 Finite element discretization

**Figure 4:**
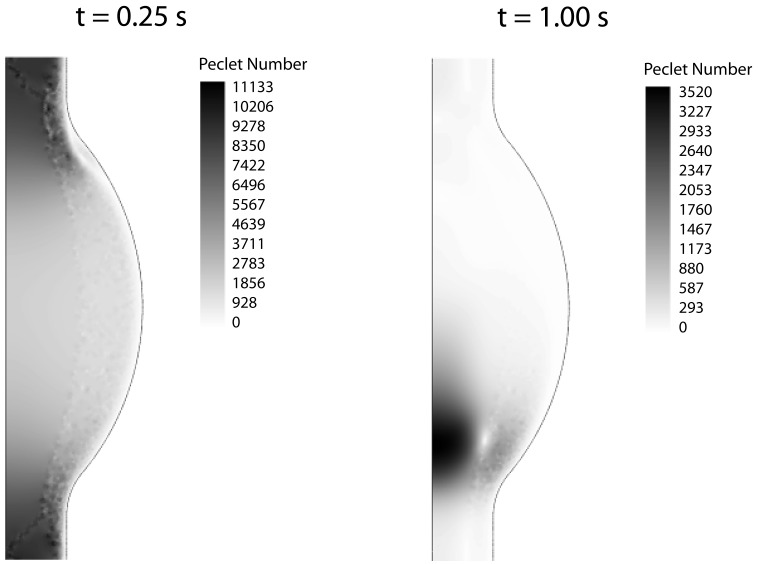
**Peclet number contours for the CDR problem at peak systole (t = 0.25 s) and late diastole (t = 1.00 s)**. In both cases the Peclet number is much larger than one in the whole domain, thus requiring numerical stabilization.

The fluid dynamical problem was discretized with Lagrange P_2_P_1_ elements, Lagrange elements with quadratic interpolation for the velocity field and linear interpolation for the pressure field, whereas linear elements were used for the CDR problem (Zienkiewicz and Taylor, 2000). The Galerkin method was used to discretize the equations, which may become unstable for an element Peclet number (Pe) larger than one, i.e., Pe = |**u**|*h*/(2D) > 1 with *h* being the mesh element size. Since both problems, fluid dynamics and CDR, are convection-dominated (Pe > 1; Figure 4), stabilization techniques have been employed.

The Navier–Stokes equations’ solution typically includes shear layers (embedded within the flow or at its boundaries) where strong gradients of the solution variables are present. The width of these layers can be smaller than the element size, and hence the solution variables cannot be resolved properly, leading to spurious oscillations (wiggles) in the solution. Galerkin least-squares (GLS) streamline diffusion combined with crosswind diffusion (*C_k_ *= 0.1) was used to stabilize the Navier–Stokes equations (Hauke, 2002; COMSOL, 2008).

The CDR problem is more challenging to stabilize. Wiggles, even of small amplitude, are not permissible in order to ensure non-negative species concentrations. Isotropic diffusion at the lowest possible level was introduced and δ*_ISO_ *= 0.1 was set (COMSOL, 2008).

##### 2.2.5.3 Time-advancing algorithm

The fluid dynamical problem and the CDR one are decoupled. Consequently, the Navier–Stokes equations were solved first and the solution (velocity vector **u**) then fed to the CDR set of equations. The Generalized-α method (Jansen et al., 2000) with the numerical damping parameter $\rho_{\infty }^{fluid}=0.75$ was used to solve the Navier–Stokes equations. The different rate constants in the chemical reactions in equations (4)–(15) define a CDR problem with temporal scales encompassing nine orders of magnitude requiring very small time steps for the non-regularized problem. The numerical parameter $\rho_{\infty }^{CDR}=0.2$ was found to be appropriate for canceling out the non-resolvable frequencies while retaining a good degree of accuracy in the computed solution.

The arising non-linear set of equations was solved using an affine invariant form of the damped Newton method (Deuflhard, 1974) together with the PARDISO direct solver (http://www.pardiso project.org/) available in COMSOL v3.5a (COMSOL AB).

All computations have been performed on a 64-bit PC equipped with 2 dual core processors Intel(R) Xeon(TM) CPU 3.40 GHz and 16 GB RAM, with Windows Server 2008 R2 Enterprise Operating System. The applied numerical parameters are summarized in Table 6.

**Table 6:** **Numerical parameters used to regularize the fluid dynamical and convection-diffusion-reaction (CDR) problems**.

*C_k_*	δ*_ISO_*	$\rho_{\infty }^{fluid}$	$\rho_{\infty }^{CDR}$
0.1	0.1	0.75	0.2

#### 2.3 Eduction scheme: The λ_2_-method

The concept of coherent vortical structures or VSs enables a precise analysis of complex flow fields. Nonetheless, the lack of an accepted mathematical definition of what constitutes a coherent vortical structure did lead to issues in its identification. The most accepted and widely used method of eduction (the process of finding a VS in a flow field) is the λ_2_-method (Jeong and Hussain, 1995). The idea behind this technique is that a vortex is a flow region with predominance of vorticity surrounding a local pressure minimum. The method is based on a reduced version of the Navier–Stokes equation for incompressible flows which ignores contributions from unsteady straining and viscous effects:

(18) $${\text{D}}^{2}+\Omega^{2}=-\frac{1}{\rho }\nabla \left(\nabla p\right).$$

This reduced version allows to analyze the pressure minima solely in relation to vortical motion; a vortex is identified as a connected region with two negative eigenvalues of **D**^2^ + **Ω**^2^. Since **D**^2^ + **Ω**^2^ is symmetric, its eigenvalues are real. Ranking the eigenvalues as λ_1_ ≥ λ_2_ ≥ λ_3_ defines the equivalent condition λ_2_ < 0 for the presence of a VS. Note that for visualization purposes, the condition λ_2_ = λ_2tr_ is typically applied, where λ_2tr_ denotes a threshold value defining the surface of the VS.

## 3 Results

### 3.1 CC model

**Figure 5:**
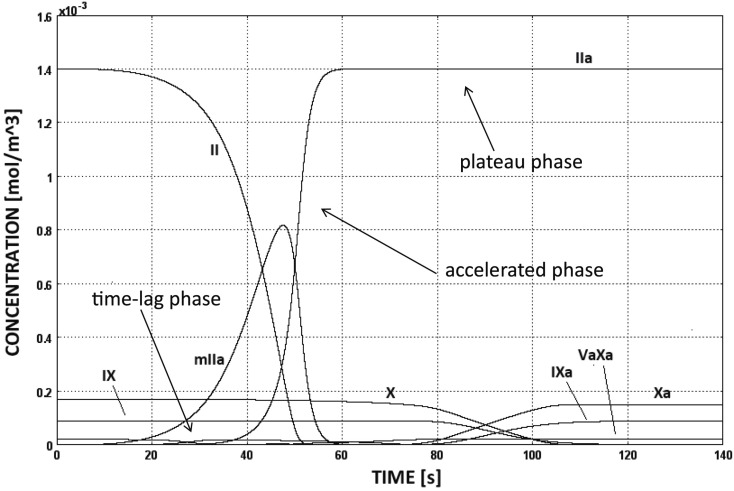
**Evolution of species’ concentrations in the CC model**. Thrombin production shows three distinct phases, i.e., time-lag, accelerated, and plateau phases. The concentrations of some chemical species are too low to be seen in the figure.

The CC is triggered as soon as TF:VIIa comes into contact with factor IX or X, i.e., at sites of subendothelial exposure, and the following evolution of species concentrations is shown in Figure 5. Initially the production of thrombin is slow (time-lag phase) and increases then rapidly due to the activation of the intrinsic pathway (accelerated phase). This boost is commonly referred to be the effect of the *positive thrombin feedback mechanism*, that is indeed the intrinsic pathway. Finally, thrombin concentration reaches the plateau value of 1.4 × 10^−3^ mol/m^3^, i.e., equal to the initial concentration of prothrombin (plateau phase).

### 3.2 Fluid-chemical model

In this section the evolution of thrombin in relation to the blood flow field is studied. Specifically, the role of VSs was investigated keeping in mind that in the limit of inviscid fluid (in the present case this is therefore still valid although to some approximation) they represent material surfaces/volumes. Consequently, VSs generated at some position tend to entrain the matter present at its formation site, i.e., VSs generated at the wall capture chemicals at that position and transport them along their trajectory.

#### 3.2.1 VSs’ dynamics and flow field

**Figure 6:**
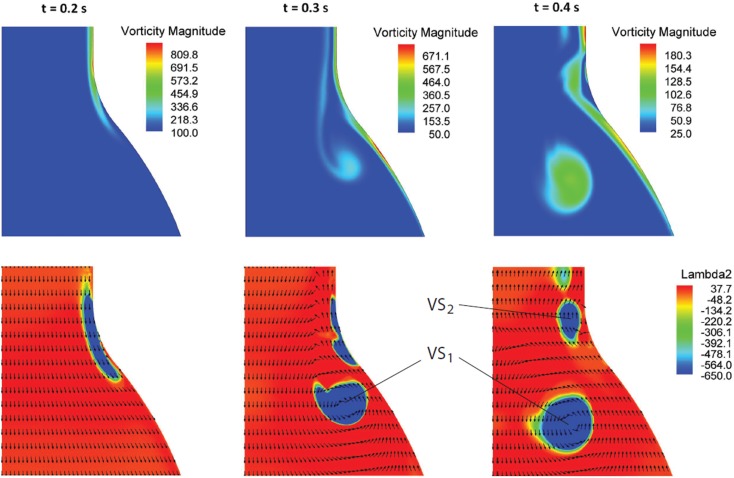
**Vorticity magnitude (s^−1^) (first row) and Vortical Structures (VSs), educed with the λ_2_-method (s^−2^), (second row) dynamics at three selected times *t* in the cardiac cycle**. The sequence highlights the formation process of the VSs (VS_1_ and VS_2_) as a result of the vorticity sheet separation and roll-up (details in the text).

**Figure 7:**
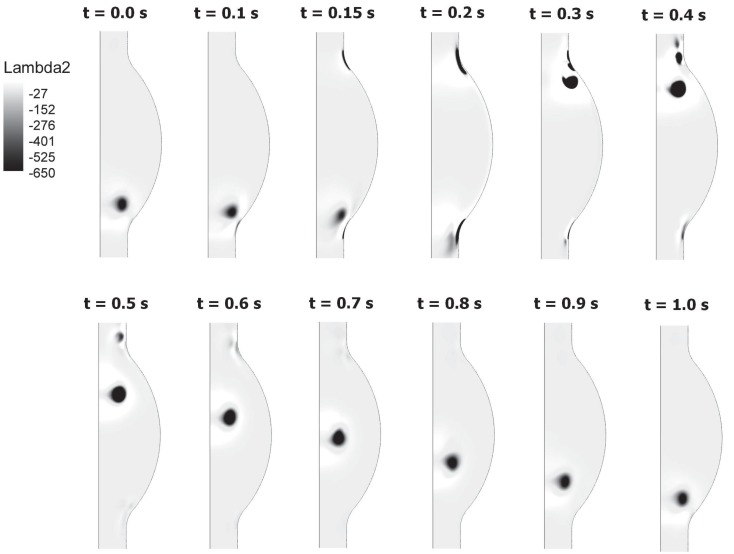
**Birth and evolution of Vortical Structures (VSs) at different times t throughout the cardiac cycle**. A vortex sheet formed at the proximal end of the AAA (t = 0.15–0.2 s) develops in a vortex (VS_1_) rotating counter-clockwise (t = 0.3 s). The vortex moves downward until it impinges on the distal contraction of the AAA. The genesis and motion of the second vortex (VS_2_) is also visible.

**Figure 8:**
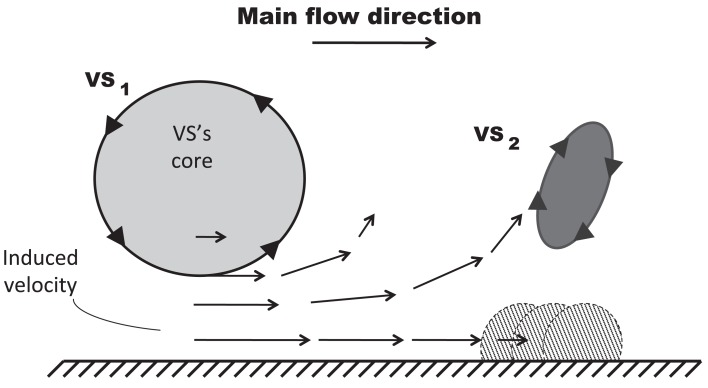
**Mechanism of VS-induced wall-vorticity accumulation and Vortical Structures (VSs) detachment**. The main VS (VS_1_), superimposed on a main flow directed from left to right, induces a velocity field in the near wall region which accumulates vorticity forming another VS (VS_2_) and, subsequently, promotes its detachment.

**Figure 9:**
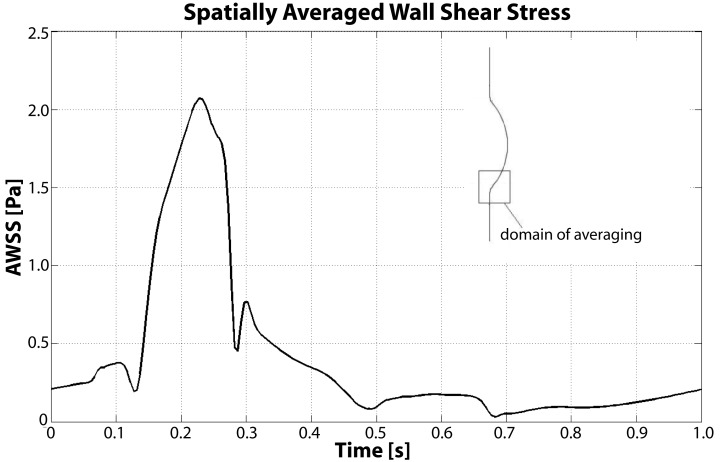
**Spatially averaged wall shear stress (AWSS) in the distal contraction during the cardiac cycle**. During Vortical Structures (VSs) impingement (between t = 0.15 s and t = 0.4 s) the averaged WSS increases more than four times with respect to the diastolic values.

At the beginning of the systolic phase (t ≈ 0.15 s), the wall shear layer separates in the expansion region giving rise to a free vorticity layer [Figure 6 (0.2 s) and Figure 7]. This free shear layer quickly increases the bending of its profile through a process driven by the velocity difference between the two sides and augmented by self-induction (Kelvin–Helmholtz instability, see, e.g., Tritton, 1988). During this process it rolls up, generating a counter-clockwise VS, from now on indicated as VS_1_, which moves away from the wall [Figure 6 (0.3 s) and Figure 7 second row]. The velocity field induced by VS_1_ generates a negative velocity gradient in the direction parallel to the wall and pointing upward, thus accumulating the diffused vorticity in the wall region. In addition, the wall-normal induced velocity component promotes the detachment of the lump of vorticity thus generated at the wall. These two effects tend synergically to cause the formation of a secondary VS [VS_2_; Figure 6 (0.3 and 0.4 s) and Figure 8]. Once detached from the wall, VS_2_ moves upstream because of the velocity field induced by VS_1_ and the influence of the mean flow [Figure 6 (0.4 s) and Figure 7]. VS_1_, on the other hand, moves downstream because of self-induction. Note that, as the computation is axisymmetric, all VSs must be seen as the trace on the computational plane of a ring vortex. Therefore VS_1_ undergoes the standard self-induction process, i.e., every section of the vortex induces downstream (because of the sign of the vorticity) velocity on the opposite section, leading to the final result of a downstream movement of the whole vortex. While moving down the lumen, VS_1_ maintains a strong identity, although the intensity is slightly reduced via viscous diffusion. When VS_1_ approaches the distal bend, it induces the generation of a shear layer which rolls up in a fashion very similar to what explained above but backward (Figure 7). This shear layer causes a remarkable wall shear stress (WSS) peak illustrated in Figure 9.

WSS is highly affected by VSs, as demonstrated by Figure 9 and our previous work (Biasetti et al., 2011); in particular a correlation between WSS and near wall VSs exists. Figure 9 shows the space-averaged WSS (AWSS) over the distal vortex impinging region. AWSS hovers below 0.5 Pa throughout the cardiac cycle due to the larger passage area caused by the aneurysmatic bulge, except when VS_1_ impinges on the wall. This happens between t = 0.1 and t = 0.4 s, where the AWSS reaches values up to 2.1 Pa. Notice that this is a spatially averaged WSS: the WSS is observed to peak at around 3.5 Pa within the averaging region. The vortex dynamics described here is coherent with experimental observations presented in Salsac et al. (2006), thus giving confirmation of the validity of the present results. For a more detailed discussion of VSs’ dynamics in AAA and a comparison between Newtonian and non-Newtonian models on VSs’ behavior the reader is referred to Biasetti et al. (2011).

#### 3.2.2 Chemical field

**Figure 10:**
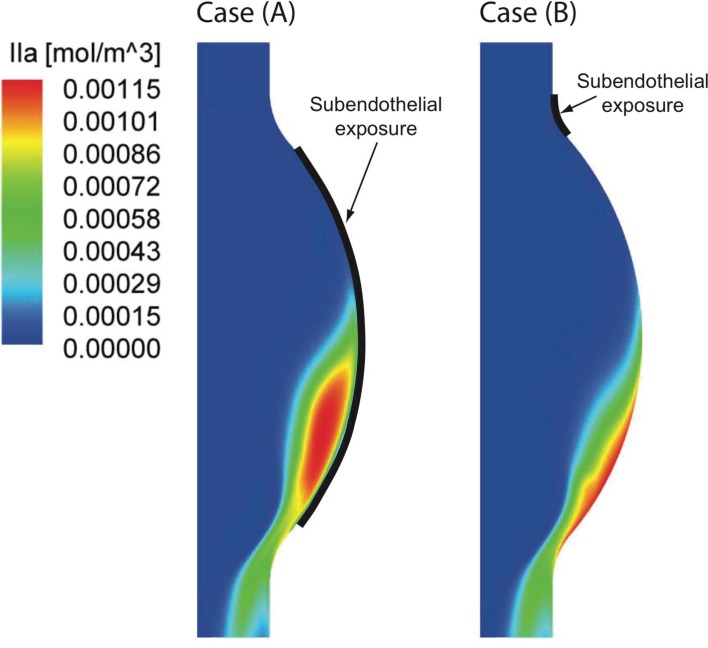
**Thrombin (IIa) distribution once the periodic state is reached in the two investigated cases**. Considering a large [Case (A)] and a small [Case (B)] subendothelial exposure (see Section 2.1) both concentration patterns are strongly shifted toward the distal Abdominal Aortic Aneurysm (AAA) region. A larger area of high thrombin concentration is found in Case (A) due to the larger endothelial damage.

**Figure 11:**
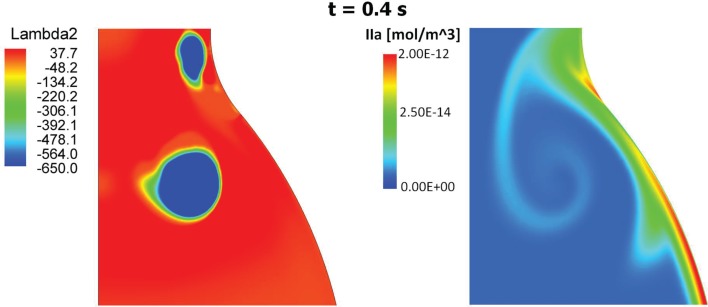
**Vortical Structures (VSs) educed with the λ_2_-method (left) and thrombin (IIa) distribution (right) at time t = 0.4 s during the cardiac cycle**. The thrombin distribution is shown using a logarithmic scale. As clearly shown, the moving VS is carrying thrombin and the characteristic counter-clockwise trail is due to the counter-clockwise rotation of the VS.

At the sites of subendothelial exposure TF:VIIa, present with the constant concentration of 1 × 10^−6^ mol/m^3^, comes into contact with factors IX and X and triggers the CC. Recall that in this case the concentration of TF:VIIa in the fluid domain is initially zero, compare with Table 4. Once triggered, the CC propagates inside the fluid and leads to thrombin production also within the lumen. After an initial transient period of about 15–20 cardiac cycles, depending on the initial subendothelial exposure area, thrombin concentration reaches a periodic solution. The combined effect of VSs shedding and recirculating region inside the aneurysmatic bulge convect thrombin to the distal AAA region in both cases of endothelial damage, i.e., case (A) and (B) (Figure 10). In the present case VS_1_ captures thrombin that is generated proximally and transports it distally due to the described VS-mediated transport phenomenon, see Section 3.2. The recirculation region, present during parts of the cardiac cycle, helps the produced chemicals to remain inside the aneurysmatic bulge. Noteworthy, VS_1_ moves downstream with an anti-clockwise rotation and creates a trail of thrombin that curls up in an anti-clockwise way (Figure 11). Fluid convection prevents thrombin over-accumulation in the distal area by washing chemicals downstream. Consequently, fluid flow might then be regarded as a “mechanical antagonist” of procoagulant accumulation.

## 4 Discussion

**Figure 12:**
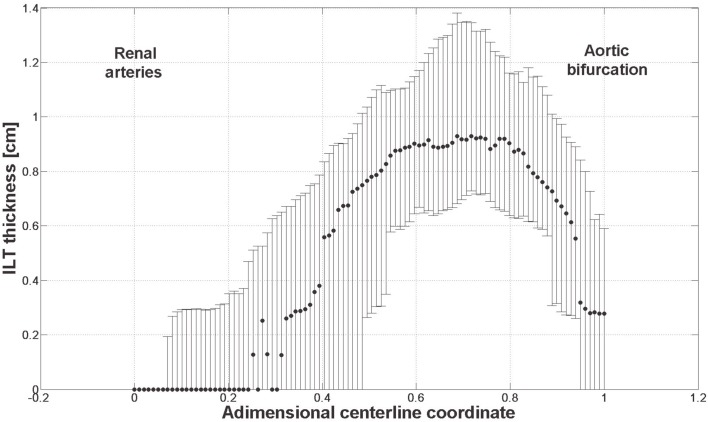
**ILT thickness measured on slices perpendicular to the centerline from 61 patients with small AAAs (<5.5 cm)**. Dots and bars denote the median and the lower and upper quartile respectively. The thickest ILT is located in the distal region of the aneurysm, at around 70% of its length starting from the renal arteries. Further details are given in Martufi et al. (submitted).

The link between biomechanical loads and biochemical signals is thought to be one of the key aspects of cardiovascular homeostasis and pathology development. Due to the complexity of the involved phenomena this link has not yet been fully elucidated. AAA is a complex pathology involving biological, mechanical, and fluid dynamical aspects and an integrated mechanochemical view may help understand its development and the role played by the ILT. Consequently, our previous fluid dynamical model (Biasetti et al., 2011) was coupled to the biochemistry allowing the study of the CC in complex flows. In earlier works (Biasetti et al., 2010, 2011) we explored the behavior of VSs and recirculating regions and we found indications that both promote favorable conditions for PLT activation, convection, and deposition at the wall in the distal AAA region. Related to this is the finding that thrombus thickness is largest in the distal portion of the aneurysmatic aorta (Figure 12). VSs capture chemicals (Figure 11), in their core and convey them until VSs’ burst (the breaking-up of the vortex), seen to happen at the distal contraction. The strong thrombin accumulation observed distally correlates with the thickness of the ILT layer, and suggests a possible correlation between fluid dynamics, biochemistry, and ILT growth. Thrombin converts soluble fibrinogen into insoluble strands of fibrin, which in turn form the clot in conjunction with platelets and other blood elements. Consequently, the accumulated thrombin in the distal AAA region might lead to an increased fibrin production enhancing ILT growth with respect to other regions. In particular, in whole blood activated with tissue factor, the production of a platelet-fibrin clot starts with thrombin concentration of approximately 2 × 10^−6^ mol/m^3^ (Mann, 2011) and during normal coagulation the concentration of thrombin can reach 5 × 10^−4^ mol/m^3^ (Wolberg and Campbell, 2008). In the cases discussed here, the thrombin concentration was observed to reach values as high as twice that reported above, i.e., ≈1 × 10^−3^ mol/m^3^.

Thrombus formation in the venous and arterial systems differs markedly. In the venous system, the low flow rates and stasis allow the accumulation of activated coagulation factors leading to the formation of thrombin mostly without platelets contribution. On the other hand, in the normal arterial circulation, flow conditions prevent this accumulation (Brass et al., 2007) and platelets play a major role in arterial thrombus formation: they accelerate thrombin formation and provide a scaffold for fibrin accumulation. In AAAs, due to pathological flow conditions involving stasis and recirculation, the normal arterial clotting process might be altered and “switch” to a mechanism similar to the one observed in the venous circulation with an enhanced accumulation of procoagulant. In addition, the convective effect of VSs tends to favor the presence of activated platelets in regions of high chemical concentrations. The combination of these effects could cause the thicker ILT observed in the distal region of the lumen – this picture being consistent with the views put forward in Swedenborg (2008), Yoshimura et al. (2011).

Below we discuss the remaining open points and limitations of the present study that should be addressed in future research.

### 4.1 Chemical model

Our model does not take into account the inhibitory processes for the reasons explained previously, but blood-borne anticoagulants from the proximal healthy arterial system might still play an important role. However to the best of the authors’ knowledge no indication has been reported in literature. Our model can then be enriched by considering this contribution too. Platelets recruited to the exposed subendothelium accumulate and cover it, blocking the source of TF:VIIa at the wall (Hathcock and Nemerson, 2005). Despite this, human thrombotic occlusion has been observed *in vivo* on a timescale of minutes (Ambrose et al., 1997) indicating that another source of procoagulant activity is available at the fluid-clot interface. Blood-borne TF has been indicated as one of the contributors (Hathcock and Nemerson, 2005). Our model does not take into account platelet accumulation at the wall allowing the continuous availability of TF:VIIa at the wall. Since the actual accretion of the ILT is not simulated in our model, the continuous availability of TF:VIIa at the wall substitutes, in first approximation, the blood-borne source of TF present at the fluid-clot interface.

To avoid numerical instabilities the diffusion coefficients used in the CDR model were increased from about 10^−10^ m^2^/s (Folie and McIntire, 1989; Xu et al., 2008) to 10^−8^ m^2^/s, which resulted in an over-diffusive solution. Since the underlying chemical field is strongly convection-dominated the influence of this numerical regularization is probably negligible, so that the general conclusions drawn here will still hold especially since lower diffusion coefficients are expected to result in even more segregation of thrombin in the near wall region.

### 4.2 ILT growth

The present model does not consider the actual ILT growth which will modify the local fluid dynamics. This alteration is difficult to predict but we expect that the growing ILT will induce VSs’ burst location to move slightly more proximally, a feature seen in patient-specific geometries (Biasetti et al., 2011). The migration of the VSs’ burst location might also be a consequence of the lost axisymmetry of the fluid domain during ILT evolution – a feature that is constrained in the present model.

It should be noted that VSs’ burst happens distally both in the present case and in patient-specific geometries (Biasetti et al., 2011), leading to the postulation that this pattern of thrombin accumulation might be found also in patient-specific cases.

### 4.3 2D-axisymmetry approximation

3D simulations of a similar flow (result not shown) reveal that in 3D the ring vortex undergoes the classical azimuthal bending instability (a waviness of its profile) which however does not modify the flow significantly. We therefore expect the 2D-axisymmetric model to provide the same results, in terms of chemical distributions, as the 3D one, with the advantage of saving computational time.

### 4.4 Parameter space

Parameters like inlet flow rate, geometry of the AAA and initial concentration of TF:VIIa can influence the distribution of the chemicals involved. A parametric study would provide important insights regarding the sensitivity of the modeled system with respect to these parameters.

### 4.5 Stabilization method

Isotropic diffusion, used to stabilize the CDR problem, is an inconsistent method and more sophisticated stabilization techniques are available (e.g., SUPG). Unfortunately, we observed that such techniques could not guarantee a non-negative concentration of the chemical species, probably due to the strong destabilizing effect of the reaction terms. For this reason, the choice of a less sophisticated but more robust method has been compulsory at the present stage.

### 4.6 Fluid-structure interaction

One limitation of the present work is the assumption of rigid wall. Despite the small motion of the aneurysm wall during the cardiac cycle (Länne et al., 1992; Long et al., 2004), and good agreement between Fluid-Structure Interaction (FSI) and rigid wall models (Wolters et al., 2005), the soft ILT tissue may deform significantly during the cardiac cycle influencing the fluid dynamics and hence the distribution of chemicals.

## Conflict of Interest Statement

The authors declare that the research was conducted in the absence of any commercial or financial relationships that could be construed as a potential conflict of interest.

## Footnotes

^1^Throughout the paper the SI unit system is used.

^2^To clarify the role of each term, and in particular of the matrix **S**, an example is given in Appendix.

## Appendix

A simple example

Consider a series of four reactions involving five species

$$\begin{array}{l} {\text{S}}_{1}\to {\text{S}}_{2},\\ 5{\text{S}}_{3}+{\text{S}}_{2}\to 4{\text{S}}_{3}+2{\text{S}}_{2},\\ {\text{S}}_{3}\to {\text{S}}_{4},\\ {\text{S}}_{4}\to {\text{S}}_{5}. \end{array}$$

The reaction rate vector **r**^T^ = [*r*_1_,*r*_2_,*r*_3_,*r*_4_] defines the reaction rates of every single reaction, i.e.

$$\begin{array}{llll} r_{1} & =k_{1}\left[{\text{S}}_{1}\right], & & \\ r_{2} & =k_{2}\left[{\text{S}}_{2}\right]{\left[{\text{S}}_{3}\right]}^{5}, & & \\ r_{3} & =k_{3}\left[{\text{S}}_{3}\right], & & \\ r_{4} & =k_{4}\left[{\text{S}}_{4}\right], & & \end{array}$$

where *k_i_*,*i *= 1, …, 4 denote the reaction rate constant of the *i*-th reaction. Consequently, the system of reactions reads

(A1) $$\frac{\text{d}}{\text{d}t}\left[\;\begin{array}{c} {\text{S}}_{1}\\ {\text{S}}_{2}\\ {\text{S}}_{3}\\ {\text{S}}_{4}\\ {\text{S}}_{5} \end{array}\right]=\underset{S}{\underset{︸}{\left[\;\begin{array}{cccc} -1 & 0 & 0 & 0\\ 1 & 1 & 0 & 0\\ 0 & -1 & -1 & 0\\ 0 & 0 & 1 & -1\\ 0 & 0 & 0 & 1 \end{array}\right]}}\left[\;\begin{array}{c} k_{1}\left[{\text{S}}_{1}\right]\\ k_{2}\left[{\text{S}}_{2}\right]{\left[{\text{S}}_{3}\right]}^{5}\\ k_{3}\left[{\text{S}}_{3}\right]\\ k_{4}\left[{\text{S}}_{4}\right] \end{array}\right],$$

where the 5 × 4 stoichiometric matrix **S** was introduced. The five rows and the four columns in **S** relate to the five species and four reactions, respectively.
